# Righting Reaction in Hemiplegic Stroke Survivors: Impact of Lower Limb Motor Paralysis Severity

**DOI:** 10.7759/cureus.82418

**Published:** 2025-04-17

**Authors:** Koki Nagai, Kazu Amimoto, Yumi Ikeda

**Affiliations:** 1 Department of Rehabilitation, Medical Corporation Sonodakai, Hanahata Rehabilitation Hospital, Tokyo, JPN; 2 Department of Physical Therapy, Graduate School of Human Health Sciences, Tokyo Metropolitan University, Tokyo, JPN; 3 Department of Rehabilitation Sciences, Sendai Seiyo Gakuin University, Miyagi, JPN

**Keywords:** compensation, hemiplegia, lower limb, postural control, righting reaction, severity, sitting balance, stroke, trunk function

## Abstract

Background and objective

The righting reaction (RR) from a laterally tilted sitting position is influenced by trunk function and walking ability; however, its relationship with the severity of lower limb motor paralysis in persons with stroke remains unclear. If the characteristics of RR response patterns are clearly identified, they may aid in determining individualized physical therapy approaches. This study aimed to clarify RR patterns according to the severity of lower limb motor paralysis and to explore their potential implications for developing individualized rehabilitation strategies.

Methods

This cross-sectional study included 51 participants with stroke requiring significant assistance in activities of daily living at a Japanese convalescent hospital. Participants were categorized into Mild (n = 16), Moderate (n = 19), and Severe (n = 16) groups based on the severity of lower limb motor paralysis. Neck, trunk, and leg angles during RR were measured. One-way ANOVA with post-hoc tests was used for intergroup comparisons.

Results

Significant differences were found in the non-paralyzed leg angle during RR toward the paralyzed side (p = 0.0003) and neck angles in both directions (paralyzed side: p = 0.0164; non-paralyzed side: p = 0.0367). The non-paralyzed leg angle was larger in the Moderate group (Mild: -3.6±6.0°, Moderate: 5.5±8.5°, Severe: -2.9±5.2°; Mild-Moderate: 95% CI: 2.6-14.1, p = 0.0011; Moderate-Severe: 95% CI: 3.1-14.7, p = 0.0022). Neck angles were larger in the Severe group than those in the Mild group (non-paralyzed side: 95% CI: 0.6-11.8, p = 0.0241; paralyzed side: 95% CI: 0.3-10.7, p = 0.0333).

Conclusions

This study demonstrated that the severity of lower limb motor paralysis significantly influences RR movement patterns in stroke survivors. Understanding RR movement patterns based on paralysis severity may aid in developing targeted rehabilitation strategies to improve postural control and functional recovery.

## Introduction

Sitting balance in persons with acute and subacute stroke is closely linked to trunk motor control [1,2]. Trunk function plays a pivotal role in stroke rehabilitation, serving as a strong predictor of activities of daily living (ADL) performance [3]. Among the components of trunk control, lateral balance stands out as a critical determinant of functional prognosis [4]. Fujino et al. demonstrated that lateral sitting balance training in an inclined position significantly improved trunk function in persons with acute stroke [5].

Numerous studies have highlighted the interplay between trunk function and motor paralysis [6,7]. Verheyden et al. examined post-stroke changes in trunk function and proposed that upper and lower limb and trunk motor paralysis follow parallel recovery trajectories, as evidenced by the temporal evolution of Fugl-Meyer Assessment scores [6]. Furthermore, the severity of upper and lower limb motor paralysis correlates with trunk function, suggesting that paralysis extent differentially impacts trunk performance [7]. The trunk impairment scale (TIS) assesses static and dynamic postural control, mobility, and coordination [8], and it has been suggested that there is a relationship with motor paralysis severity. Previous studies have reported a relationship between walking ability, speed, and independence and TIS [8]. It is thought that postural control may differ depending on the degree of lower limb motor paralysis [6,7]. In this study, we focused on the effect of lower limb motor paralysis on lateral sitting balance, which is part of trunk function. However, its relationship with lateral sitting balance remains unclear. If lower limb motor paralysis affects lateral sitting balance, detailed evaluation and tailored interventions may be necessary.

The TIS [8], trunk control test [9], and function in sitting test (FIST) [10] have been established as methods for assessing trunk function and sitting balance in persons with stroke. The FIST assesses individuals with low physical function, whereas TIS and TCT may have floor effects in severe cases. Moreover, these interval-scale assessments may lack sensitivity to subtle changes. Given the need for a safe and accurate evaluation of lateral sitting balance in severe hemiplegia, we developed a method to quantify the righting reaction (RR), which has demonstrated high reliability [11]. In this study, RR refers not to a traditionally "reflexive" phenomenon but rather to a "voluntary" action of repositioning oneself upright against gravity from a laterally tilted sitting position, defined as behavioral verticality and trunk control [12]. This method quantifies RR angles [11], with a minimal clinically important difference from 2 to 5° reported in severe subacute stroke cases [13]. This method incorporates protective measures, including walls on the patient’s left, right, and back sides, to minimize fall risks, even in severe stroke cases. Other previous studies quantitatively assessed dynamic sitting balance in response to sudden lateral tilt stimuli using an unstable seesaw-like platform in ambulatory patients [14]. Additionally, previous research has shown that intervention with an unstable board for stroke survivors enhanced the trunk RR angle while reducing compensatory rotation of the non-paralyzed hip joint [13]. Moreover, walking ability influences RR patterns; non-independent walkers exhibited increased neck compensation during sudden lateral tilts [14]. Trunk function and walking ability likely influence RR patterns, but their direct relationship with motor paralysis severity remains unclear. Examining this connection is crucial for understanding postural control in a person with a stroke. Analyzing RR patterns may help clarify their role in postural stability and rehabilitation strategies.

While motor paralysis and hemiplegia severity are known to affect trunk function, their specific relationship with lateral sitting balance remains unexplored. Particularly, severe hemiplegia is often associated with reduced physical activity, which may lead to disuse muscle atrophy [15], further influencing postural control. If RR response patterns are found to depend on motor paralysis severity, they may inform physical therapy approaches to improve lateral sitting balance in persons with severe stroke. We hypothesized that the severity of lower limb motor paralysis influences RR angle and response patterns, with RR primarily involving trunk movements in the Mild group, while compensatory use of the neck and lower limbs would be more pronounced in the Moderate and Severe groups. Identifying these patterns may help tailor physical therapy interventions: the Mild group may benefit from RR tasks on larger inclines, the Moderate group from trunk activation strategies that limit compensation, and the Severe group from interventions targeting disuse muscle atrophy. This study aimed to clarify RR characteristics by the severity of lower limb motor paralysis and propose individualized therapy strategies.

## Materials and methods

Study design

This is a single-center, cross-sectional study with blinded assessors, conducted in adherence with the Strengthening the Reporting of Observational studies in Epidemiology (STROBE) guidelines [16].

Participants

This cross-sectional study included participants admitted to a convalescent rehabilitation hospital in Tokyo, Japan, between December 2020 and August 2023. G*power (version 3.1.9.7; Heinrich Heine University, Dusseldorf, Germany) was used to determine the sample size [17]. The sample size was calculated with an alpha error of 0.05, a power of 0.80, and an effect size of 0.25 [18], resulting in a required total of 159 participants (53 per group). Power was calculated by the total sample and the effect size. Based on a previous study [13], power for the primary outcome measure (RR evaluation) was calculated during recruitment. Enrollment ceased once power exceeded 0.95, ensuring sufficient detection capability. The inclusion criteria were as follows: ability to understand instructions, ability to sit with supervision or independently for at least one minute, a functional ambulation categories (FAC) score of less than three points [19], and a modified Rankin scale (mRS) score of four or five points [20]. These participants had poor walking function and required significant assistance with ADL. Participants were excluded if they had a history of orthopedic disease of the lower limbs, psychiatric illness, or orthostatic hypotension in either the sitting or standing position. Screening assessments were conducted during physical therapy sessions, and patients who met the inclusion criteria were included in the study. Therefore, the timing of evaluation varied among participants and was not standardized to a specific time. Participants were classified into three groups using the Brunnstrom recovery stages (BRS) of the lower limb (the Mild (BRS V, VI), Moderate (BRS III, IV), and Severe (BRS I, II) groups) [21] based on the severity of lower limb motor paralysis, considering the impact on postural control. A similar classification method has been employed in previous studies [22].

This study was conducted in accordance with the Declaration of Helsinki and approved by the Ethics Committee of Sonodakai (approval number no. 122) and the Research Ethics Committee of Tokyo Metropolitan University Arakawa Campus (approval no. 22054). Written and verbal consent was obtained from all participants before they participated in the study.

Assessment

Data Collection

Data on age, sex, days from onset to measurement, diagnosis, and paralyzed side were collected from participants’ medical records. Assessments of physical functions were also conducted, including the BRS [21], superficial and deep sensory tests [23], and the FAC (walking ability) [19]. The trunk function was evaluated using the TIS [8], and ADL was evaluated using the functional independence measure (FIM) [24]. Trained physical therapists evaluated the BRS, sensory test, FAC, TIS, and FIM.

Primary Outcome

**Figure 1:**
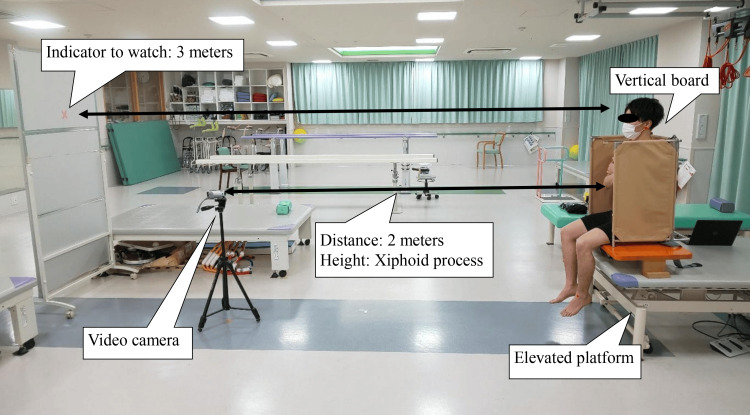
Environment setting for the righting reaction assessment. A video camera (Everio; JVC Kenwood, Kanagawa, Japan) was positioned 2 m from the VB at the participant’s xiphoid process level. During the evaluation, the participants were instructed to gaze at the target 3 m away.

As the primary outcome, the lateral sitting balance was evaluated using the RR angle [11] and center of pressure (COP) displacement distance [13]. Since this study targeted persons with stroke who required significant assistance with ADL, a method was used to minimize the risk of falls. A vertical board (VB) with the back and both side walls was placed on the platform, which can move upward or downward. A seat pressure distribution sensor device (SR Soft Vision; Sumitomo Riko Co., Ltd., Aichi, Japan) featuring 256 sensors and a sampling frequency of 20 Hz was placed on the seating platform. Participants sat on the platform, which was tilted laterally at a 10° angle in the frontal plane, with their feet off the ground. Participants were instructed to "sit up straight without twisting" and perform RR while video-recorded for 10 seconds. The RR task was performed with a tilt direction in the following sequence: non-paralyzed side, paralyzed side, paralyzed side, and non-paralyzed side. “Paralyzed side tilt” is defined as inclining the sitting board toward the paralyzed side. A video camera (Everio; JVC Kenwood, Kanagawa, Japan) was positioned 2 m from the VB at the level of the participant’s xiphoid process (Figure 1). During the evaluation, the participants were instructed to gaze at the target 3 m away (Figure 1).

**Figure 2:**
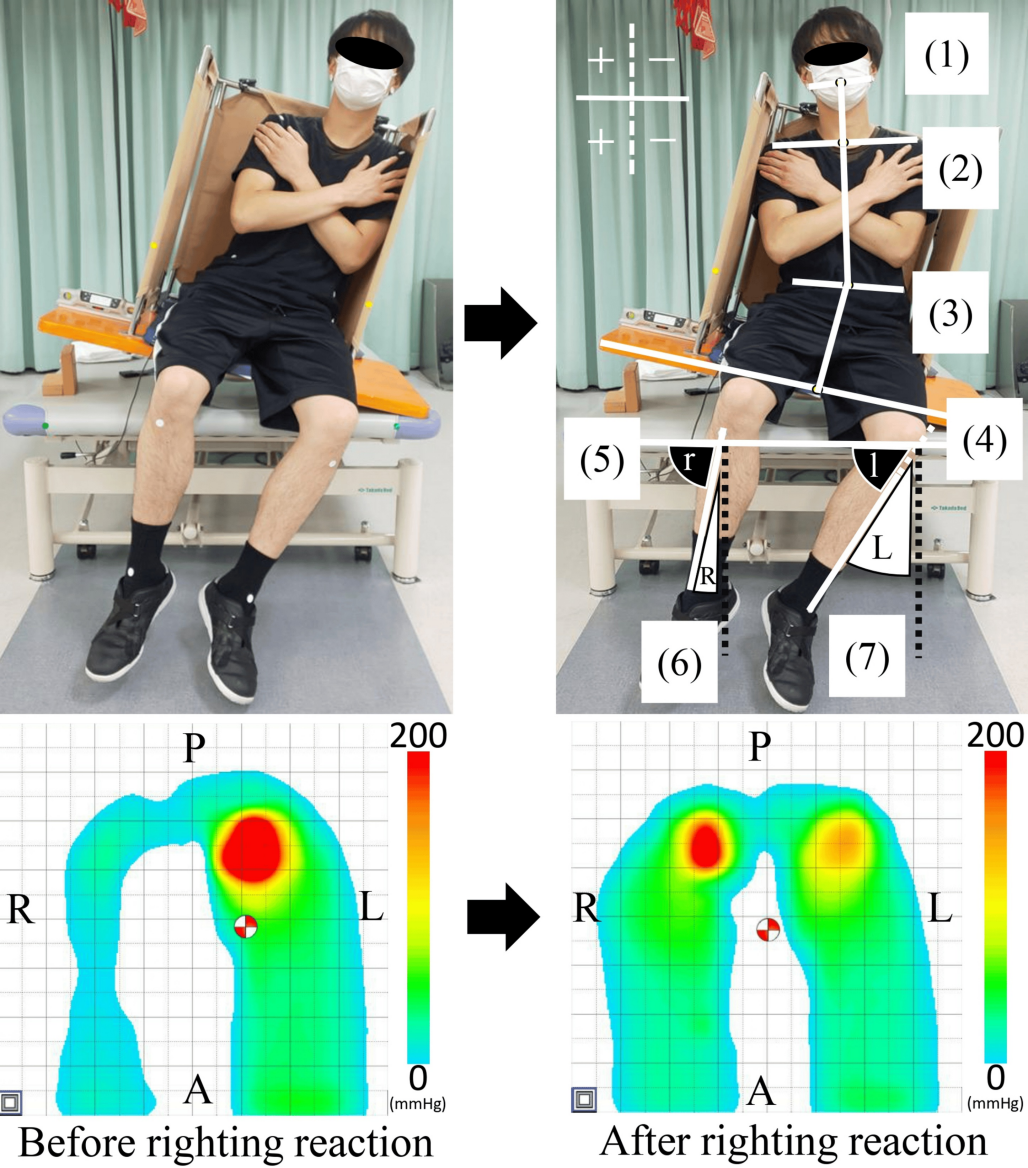
Calculation of the angle of the righting reaction and the center of pressure displacement distance (e.g., righting reaction toward non-paralyzed side in left hemiplegia). Angle measurement (upper): (1) midpoint of the line connecting the two earlobes; (2) midpoint of the line connecting the two anterior acromial surfaces; (3) midpoint of the line connecting between anterior superior iliac spine on both sides; (4) midpoint of the upper edge of the vertical board; (5) line connecting the two frontal points of the elevated platform; (6) line connecting the right tibial tuberosity to the midpoint of the line connecting the right medial and lateral malleoli; (7) line connecting the left tibial tuberosity to the midpoint of the line connecting the left medial and lateral malleoli. Angle of the neck: between the line connecting (1), (2), and (3); angle of the trunk: between the line connecting (2), (3), and (4); angle of the right lower leg (R): 90 minus “r” (the angle between [5] and [6]); angle of the left lower leg (L): 90 minus “l” (the angle between [5] and [7]). Positive and negative values were defined based on the reference dotted line on the image; positive values indicated movement toward the non-paralyzed side and negative values indicated movement toward the paralyzed side. The center of pressure displacement distance (lower): red and white circles indicate the centers of pressure. Number of sensors, 256; sampling frequency, 20Hz; A = anterior; P = posterior; R = right; L = left. The color bar indicate seating pressure (mmHg) during the righting reaction task.

Markers were attached bilaterally to each participant’s auricular lobules, anterior acromial surfaces, anterior superior iliac spines, tibial tuberosities, medial and lateral malleoli, and two points in front of the elevated platform, for a total of 12 locations. Video data were converted to still images using the Free Video to JPG Converter (DVDVideoSoft Ltd., Roseau, Dominican Republic; https://www.dvdvideosoft.com/products/dvd/Free-Video-to-JPG-Converter.htm). Two images were imported into ImageJ for analysis: one showing the tilted sitting position and another taken during RR. The RR angles of the neck, trunk, and lower legs were also calculated. Lines connecting the respective anatomical markers were plotted using ImageJ, as illustrated in Figure 2. Based on the angles of these lines, the direction of the RR was defined as positive (Figure 2).

The COP displacement distance during the RR was obtained using the seat pressure gauge analysis software. It was calculated as the difference between the COP at rest (in the tilted sitting position) and the COP during RR. Positive and negative values were defined according to the seat pressure gauge, with positive values indicating movement in the direction of the RR (Figure 2). RR angle and COP displacement distance was performed by two independent therapists who were blinded to group allocation.

Statistical analysis

Quantitative variables are described as mean ± standard deviation and median (interquartile range) for parametric and non-parametric variables, respectively. There were no missing values ​​for each outcome. The Shapiro-Wilk test was used to determine normality. Categorical variables are expressed as the number of participants (%). Intergroup comparisons were performed using a one-way analysis of variance (ANOVA). Comparisons were made using the chi-square test for sex, paralyzed side, and type of diagnosis. When significant differences were found in the ANOVA, further comparisons were conducted using the Bonferroni method as a post-hoc test. Statistical analyses were performed using Statistical Product and Service Solutions (SPSS, version 26; IBM SPSS Statistics for Windows, Armonk, NY), with a significance level of 5%.

## Results

**Figure 3:**
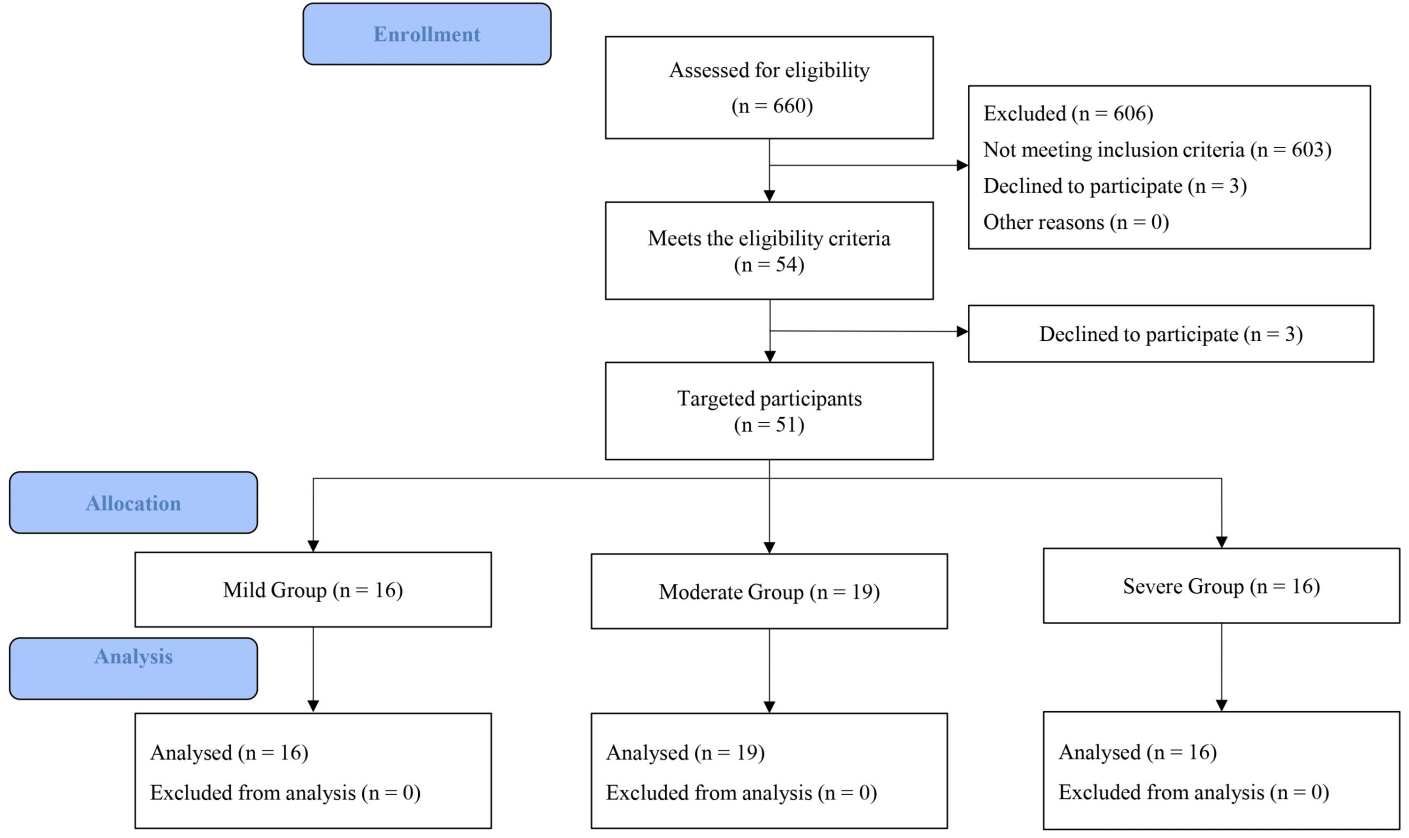
Participant enrollment and selection flow diagram. The 51 targeted participants were divided into three groups based on the severity of hemiplegia, assessed using the Brunnstrom Recovery Stage (BRS) (participants were assigned to the Mild (BRS V, VI), Moderate (BRS III, IV), and Severe (BRS I, II) groups, respectively).

Out of 660 initially enrolled participants, 603 did not meet the inclusion criteria, and six declined, leaving a total of 51 participants who met the inclusion and exclusion criteria (Figure 3). Recruitment was halted after 51 participants, as power analysis for the RR angle of the non-paralyzed lower limb reached 0.97 (Table 1) [13]. The 51 participants were divided into three groups based on the severity of hemiplegia, assessed using the BRS of the lower limb (16 (31.4%), 19 (37.3%), and 16 (31.4%) participants were assigned to the Mild (BRS V, VI), Moderate (BRS III, IV), and Severe (BRS I, II) groups, respectively (Figure 3)) [21]. This classification method has also been employed in previous studies [22]. No adverse events occurred during the RR assessment.

**Table 1:** Power calculation of outcomes. Abbreviations: RR, righting reaction; COP, center of pressure

Outcome parameters	Power (1-β)
RR angle toward the non-paralyzed side	
Neck	0.76
Trunk	0.15
Paralyzed lower leg	0.39
Non-paralyzed lower leg	0.17
RR angle toward the paralyzed side	
Neck	0.65
Trunk	0.28
Paralyzed lower leg	0.34
Non-paralyzed lower leg	0.98
The COP displacement distance during the RR	
Toward non-paralyzed side	0.52
Toward paralyzed side	0.14

Participants’ demographic data are presented in Table 2. The mean age of the participants was 69.1±13.5 years, and 58.8% were men. No significant differences were observed between the groups (p > 0.05). The TIS scores were 12.4±4.4 points, 10.7±4.2 points, and 7.1±3.0 points in the Mild, Moderate, and Severe groups, respectively. Regarding FIM, the FIM-M scores were 38.2±8.5 points, 31.1±8.1 points, and 23.8±7.8 points in the Mild, Moderate, and Severe groups, respectively. The FIM-Total scores were 57.6±11.3 points, 48.1±9.7 points, and 40.4±11.4 points in the Mild, Moderate, and Severe groups, respectively. ANOVA on TIS, FIM-M, and, FIM-Total scores revealed significant differences between groups (TIS: F (2,118) = 7.68, p = 0.0013, 95% CI: 8.9-11.3, η^2^ = 0.24, FIM-M: F (2,833) = 12.59, p < 0.0001, 95% CI: 28.3-33.7, η^2^ = 0.34, FIM-Total: F (2,1186) = 10.26, p = 0.0002, 95% CI: 45.2-52.1, η^2^ = 0.30).

**Table 2:** Patients’ demographic data in each the severity of lower limb motor paralysis. Abbreviations: FAC, Functional Ambulation Categories; 95% CI, 95% confidence interval. ^a^Continuous data are expressed as means ± standard deviations. ^b^Median (interquartile range). ^c^P-values obtained from one-way analysis of variance. ^d^P-values obtained from chi-square tests. ^e^P-values obtained from Kruskal-Wallis tests. ^f^Statistically significant difference (p < 0.05).

	Mild*^a^*	Moderate*^a^*	Severe*^a^*	Test statistic value	P-value	95% CI	Effect size
	(n = 16)	(n = 19)	(n = 16)				
Age (years)	65.6 ± 14.2	69.5 ± 12.2	72.1 ± 14.4	F (2, 168) = 0.92	0.4067*^c^*	65.3-72.9	η^2^ = 0.10
Sex (males/females)	8/8	10/9	9/7	Chi-square value = 1.25	0.5764*^d^*	-0.15- 0.22	V = 0.04
Paralyzed side (right/left)	7/9	6/13	5/11	Chi-square value = 0.73	0.6941*^d^*	-0.10-0.27	V = 0.08
Days from stroke onset (days)	62.8 ± 36.4	74.1 ± 35.1	79.3 ± 39.5	F (2, 1137) = 0.83	0.4412*^c^*	61.8-82.5	η^2^= 0.04
Stroke etiology (infarction/ hemorrhage)	7/9	13/6	8/8	Chi-square value = 2.36	0.3071*^d^*	-0.03-0.34	V = 0.15
Sensory status (Normal/Mild/Moderate/Severe/Loss)							
Superficial sensation	3/8/2/2/1	3/10/2/2/2	2/6/4/2/2	Chi-square value = 2.35	0.9684*^d^*	-0.03-0.34	V = 0.15
Deep sensation	6/5/2/2/1	3/8/3/3/2	2/7/3/2/2	Chi-square value = 2.02	0.9178*^d^*	-0.02-0.21	V = 0.01
*^b^*FAC (points)	1 (1-1)	1 (0-1)	0 (0-1)	F (2, 1) = 2.96	0.0727*^e^*	0.63-1.01	η^2^ = 0.03
Trunk Impairment Scale (points)							
Total	12.4 ± 4.4	10.7 ± 4.2	7.1 ± 3.0	F (2, 118) = 7.68	0.0013*^cf^*	8.9-11.3	η^2^ = 0.24
Functional Independent Measure (points)							
Motor	38.2 ± 8.5	31.1 ± 8.1	23.8 ± 7.8	F (2, 833) = 12.59	<0.0001*^cf^*	28.3-33.7	η^2^ = 0.34
Cognitive	19.4 ± 6.6	17.1 ± 4.5	16.6 ± 6.6	F (2, 36) = 1.03	0.3667*^c^*	16.0-19.3	η^2^ = 0.04
Total	57.6 ± 11.3	48.1 ± 9.7	40.4 ± 11.4	F (2, 1186) = 10.26	0.0002*^cf^*	45.2-52.1	η^2^ = 0.30

The results of the RR angle and COP displacement distance are presented in Table 3. The neck angle was 11.3±5.7°, 12.3±6.7°, and 17.5±6.6° in the Mild, Moderate, and Severe groups during RR toward the non-paralyzed side, and was 10.9±6.6°, 13.1±6.7°, and 16.4±3.7° in the Mild, Moderate, and Severe groups during RR toward the paralyzed side, respectively. ANOVA revealed significant differences in the RR angle of the neck between groups, regardless of the RR direction (RR to non-paralyzed side: F (2, 182) = 4.48; p = 0.0164, 95%CI: 11.7-15.5, η^2^ = 0.16; RR to paralyzed side: F (2, 123) = 3.54, p = 0.0367, 95% CI: 11.7-15.1, η^2^ = 0.13). In addition, the RR angle of the non-paralyzed lower leg was -3.6±6.0° in the mild group, 5.5±8.5° in the moderate group, and -2.9±5.2° in the severe group in the RR toward the paralyzed side. Regarding the RR to the paralyzed side, an ANOVA revealed a significant difference in the RR angle of the non-paralyzed lower leg between groups (F (2, 446) = 9.53, p = 0.0003, 95% CI: -2.2-2.2, η^2^ = 0.28). COP displacement distance was not significantly different between groups, regardless of RR direction (p > 0.05).

**Table 3:** Results of one-way analysis of variance for righting reaction. Abbreviations: RR, righting reaction; COP, center of pressure; 95% CI, 95% confidence interval. *^a^*Continuous data are expressed as means ± standard deviation. ^b^P-values obtained from one-way analysis of variance. ^c^Statistically significant difference (p < 0.05).

	Mild*^a^*	Moderate*^a^*	Severe*^a^*	F-value	P-value^*b*^	95% CI	Effect size
	(n = 16)	(n = 19)	(n = 16)				
RR angle toward the non-paralyzed side (°)							
Neck	11.3 ± 5.7	12.3 ± 6.7	17.5 ± 6.6	4.48 (2, 182)	0.0164*^c^*	11.7-15.5	η^2^ = 0.16
Trunk	13.0 ± 4.7	12.6 ± 4.3	11.3 ± 4.1	0.66 (2, 12)	0.5231	11.2-13.5	η^2^ = 0.03
Paralyzed lower leg	-0.8 ± 5.5	0.9 ± 5.8	-2.1 ± 3.3	1.52 (2, 39)	0.2288	-2.0-0.8	η^2^ = 0.07
Non-paralyzed lower leg	0.9 ± 7.6	4.7 ± 10.6	-0.8 ± 5.5	1.98 (2, 138)	0.1502	-0.5-4.1	η^2^ = 0.03
RR angle toward the paralyzed side (°)							
Neck	10.9 ± 6.6	13.1 ± 6.7	16.4 ± 3.7	3.54 (2, 123)	0.0367*^c^*	11.7-15.1	η^2^ = 0.13
Trunk	14.3 ± 6.9	12.5 ± 6.1	10.4 ± 3.8	1.83 (2, 61)	0.1710	10.8-14.0	η^2^ = 0.07
Paralyzed lower leg	0.2 ± 6.8	1.4 ± 4.2	-1.7 ± 3.5	1.62 (2, 41)	0.2082	-1.3-1.4	η^2^ = 0.06
Non-paralyzed lower leg	-3.4 ± 6.0	5.5 ± 8.5	-2.9 ± 5.2	9.53 (2, 446)	0.0003*^c^*	-2.2-2.2	η*^2^* = 0.28
The COP displacement distance during the RR (mm)							
Toward non-paralyzed side	6.00 ± 9.61	4.39 ± 8.80	3.89 ± 10.15	0.22 (2, 39)	0.8045	2.2-7.3	η^2^ = 0.01
Toward paralyzed side	4.06 ± 9.40	2.38 ± 8.30	0.88 ± 8.24	0.54 (2, 81)	0.5864	0.1-4.8	η^2^ = 0.01

The post-hoc test is shown in Table 4. The Severe group had a significantly larger neck RR angle than the Mild group (RR to the non-paralyzed side: 95% CI: 0.6-11.8, p = 0.0241, d = 1.01; RR to the paralyzed side: 95% CI: 0.3-10.7, p = 0.0333, d = 1.07). The Moderate group had a significantly larger non-paralyzed lower leg RR angle than the Mild and Severe groups (Mild-Moderate: 95% CI: 2.6-14.1, p = 0.0011, d = 1.23; Moderate-Severe: 95% CI: 3.1-14.7, p = 0.0022, d = 1.23). In contrast, no significant differences were observed between groups in the RR of the non-paralyzed lower leg to the non-paralyzed side or in the trunk and paralyzed lower leg in either direction (p > 0.05). The TIS score was significantly higher in the Mild group than in the Severe group (p = 0.0013, 95% CI: 1.9-8.7, d = 1.44), while the Moderate group had significantly higher scores than the Severe group (p = 0.0302, 95% CI: 0.3-6.7, d = 1.00). The FIM-M scores were significantly higher in the Mild group than in the Moderate and Severe groups, and in the Moderate group than in the Severe group (Mild-Moderate: p = 0.0378, 95% CI: 0.5-14.2, d = 0.86; Mild-Severe: p < 0.0001, 95% CI: 7.3-21.6, d = 1.72, Moderate-Severe: p = 0.0389, 95% CI: 0.3-14.0, d = 0.92). Similarly, the FIM-Total scores were significantly higher in the Mild group than in the Moderate and Severe groups (Mild-Moderate: p = 0.0378, 95% CI: 0.4-18.5, d = 0.90; Mild-Severe: p = 0.0001, 95% CI: 7.8-26.6, d = 1.51). No significant difference was observed between the moderate and severe groups (p > 0.05).

**Table 4:** Post-hoc comparisons of righting reaction, trunk function, and ADL ability by the severity of lower limb motor paralysis. Abbreviations: RR, righting reaction; 95% CI, 95% confidence interval. *^a^*P-values obtained from post hoc tests, performed using the Bonferroni method. *^b^*Statistically significant difference (p < 0.05).

	Intergroup comparison	P-value*^a^*	95% CI		Effect size
RR angle toward the non-paralyzed side					
Neck	Mild-Moderate	1.0000		-4.3-6.4	d = 0.17
	Mild < Severe	0.0241*^b^*		0.65-11.8	d = 1.01
	Moderate-Severe	0.0587		-0.5-10.6	d = 0.78
RR angle toward the paralyzed side					
Neck	Mild-Moderate	0.8314		-2.8-7.6	d = 0.33
	Mild < Severe	0.0333*^b^*		0.3-10.7	d = 1.07
	Moderate-Severe	0.3143		-1.7-8.3	d = 0.63
Non-paralyzed leg	Mild < Moderate	0.0011*^b^*		3.1-14.7	d = 1.22
	Mild-Severe	1.0000		-5.5-6.5	d = 0.09
	Moderate>Severe	0.0022*^b^*		2.7-14.1	d = 1.22
Trunk Impairment Scale					
Total	Mild-Moderate	0.5795		-5.8-1.5	d = 0.41
	Mild > Severe	0.0011*^b^*		1.9-8.7	d = 1.44
	Moderate > Severe	0.0302*^b^*		0.3-6.7	d = 1.00
Functional Independent Measure					
Motor	Mild > Moderate	0.0330*^b^*		0.5-14.2	d = 0.86
	Mild > Severe	<0.0001*^b^*		7.3-21.6	d = 1.72
	Moderate > Severe	0.0389*^b^*		0.3-14.0	d = 0.92
Total	Mild > Moderate	0.0378*^b^*		0.4-18.5	d = 0.90
	Mild > Severe	0.0001*^b^*		7.8-26.6	d = 1.51
	Moderate-Severe	0.1178		-1.3-16.8	d = 0.73

## Discussion

This study is the first to examine the RR from a laterally tilted sitting position in relation to the severity of lower limb motor paralysis. The Moderate group exhibited a larger RR angle in the non-paralyzed lower limbs than the Mild and Severe groups, while the Severe group showed a larger neck RR angle. These findings indicate that RR response patterns vary depending on the severity of lower limb motor paralysis.

Despite differences in trunk function among the groups, no significant differences were observed in the trunk RR angle or COP displacement distance. Previous studies have correlated reach distance with COP displacement in seated reaching tasks [25]. Given that a previous study [13] reported increased trunk RR angles and COP displacements following trunk training on an unstable board, we anticipated larger trunk RR angles and COP displacements in the Mild group; however, our findings did not support this hypothesis. This discrepancy may stem from compensatory strategies: the Moderate group relied on increased non-paretic lower limb movement, while the Severe group compensated with greater neck involvement. In contrast, the Mild group exhibited smaller RR angles in the neck and lower limbs, suggesting more efficient RR, which was characterized by minimal compensation and energy-efficient postural control. Excessive external hip rotation or neck compensation may indicate impaired trunk function, particularly in severe hemiplegia.

TIS scores in this study (Moderate: 10.7±4.2 points, Severe: 7.1±3.0 points) indicate impaired trunk function in the Moderate and Severe groups, consistent with studies showing motor paralysis severity impacts trunk function [6,7]. However, the trunk RR angle and COP displacement remained comparable across groups, possibly due to compensation strategies. The Moderate group exhibited greater external hip rotation of the non-paralyzed leg during RR toward the paralyzed side. In a person with a stroke, motor unit recruitment is particularly impaired in the paralyzed-side trunk muscles, reducing muscle activity compared to healthy individuals [26]. The trunk RR angle may decrease during RR toward the paralyzed side due to reduced muscle activity in the paralyzed side trunk muscles. Although TIS scores did not significantly differ between the Mild (12.4±4.4) and Moderate (10.7±4.2) groups, the Moderate group had lower scores, suggesting reduced trunk function. To compensate, participants in this study likely relied more on the non-paralyzed lower limb. This finding is consistent with previous research, indicating that individuals with stroke prioritize the non-paralyzed lower limb when recovering from lateral balance perturbations in standing [27]. Similarly, RR toward the paralyzed side in this study may have induced lateral resistance on the paralyzed side during non-paralyzed side tilting, potentially leading to excessive hip external rotation on the non-paralyzed side. Therefore, we considered that larger compensation by externally rotating the hip on the non-paralyzed side was required by the Moderate group when performing RR toward the paralyzed side.

In contrast, the Severe group primarily relied on neck muscles regardless of RR direction. Previous research has demonstrated a relationship between physical activity and function in persons with stroke [15]. Disuse muscle atrophy is common in severe hemiplegia with low physical activity levels [15]. In this study, the Severe group exhibited significantly lower ADL ability, as reflected in the FIM motor scores (Mild: 38.2±8.5, Moderate: 31.1±8.1, Severe: 23.8±7.8). This suggests that decreased ADL ability contributed to reduced physical activity, potentially leading to disuse muscle atrophy in both lower limbs. Similar to the Moderate group, the Severe group also had impaired trunk function, necessitating compensatory postural control during RR. However, unlike the Moderate group, the progression of disuse muscle atrophy in the lower limbs may have made lower limb compensation difficult. Consequently, the Severe group primarily relied on the neck to compensate for impaired trunk function and reduced lower limb muscle output, leading to a larger neck RR angle. Therefore, it was assumed that the Severe group, who likely had poor ADL ability and decreased physical activity, developed disuse atrophy in both the lower limb muscles, leading to their primary reliance on neck muscles for compensation.

These findings emphasize the possibility of considering RR response patterns in rehabilitation and can help devise tailored approaches based on compensation mechanisms. If a patient primarily compensates with the neck, whole-body disuse muscle atrophy may be present. Initial training should prioritize stabilizing the paretic side using a knee ankle foot orthosis, followed by gait training and whole-body exercises. If RR evaluation later indicates a shift from neck to non-paralyzed lower limb compensation, this suggests improved muscle function but persistent trunk dysfunction. In such cases, training with RR should limit excessive non-paralyzed hip compensation or adjust task difficulty to minimize compensatory movements. Trunk training strategies reported in previous studies [5,13,28,29] may also be beneficial depending on the patient's condition. Future studies should explore whether interventions targeting these compensatory patterns enhance postural control and functional recovery in persons with stroke.

Limitations

This study has several limitations. First, as a cross-sectional study, it cannot establish causal relationships between RR response patterns and functional outcomes. Future longitudinal or interventional studies are needed to clarify whether RR training directly improves trunk function. Second, this study was conducted at a single rehabilitation center, which may limit the generalizability of the findings. Multi-center studies with a larger and more diverse population are necessary to confirm the applicability of our results. Third, while RR measurements are highly reliable [11], this study did not assess joint range of motion, brain activity, or trunk muscle activation, which could provide further insight into the mechanisms underlying RR responses. Further research incorporating electromyographic and motion analyses is needed to clarify this aspect. Additionally, RR may have been influenced by visual input; however, a subjective visual vertical (SVV) assessment was not conducted due to equipment limitations. Future studies should integrate neural, SVV, and musculoskeletal assessments to better understand their impact on RR patterns. Fourth, RR evaluation in this study was based on still images and did not involve real-time dynamic assessment. Future research may need to explore alternative methods, such as assessing response patterns instead of angular measurements, to enable real-time evaluation. Fifth, this study utilized a VB for RR measurement with safety considerations. While no participants collided with the VB walls, their presence may have restricted natural movement patterns and limited the full expression of compensatory strategies. Although the VB provides standardized conditions for assessment, further studies are needed to determine whether alternative wall-free measurement setups would allow a more comprehensive assessment of the RR response while minimizing the risk of falls. Finally, while the RR assessment system used in this study offers high safety and quantifiability, it may have limited feasibility in routine clinical settings, as the standardized setup and equipment (e.g., vertical board and pressure sensors) are not commonly available in many clinics. Further research is needed to develop simplified and widely applicable assessment tools.

## Conclusions

In conclusion, this study demonstrated that the severity of lower limb motor paralysis significantly influences RR movement patterns in stroke survivors, inducing varying compensatory mechanisms. Understanding the different compensatory RR response patterns may help therapists develop individualized approaches to improve lateral sitting balance, highlighting its clinical significance. These insights emphasize the clinical value of RR evaluation as a novel assessment tool that complements conventional measures like the TIS.
